# Abstracts from German Journals

**Published:** 1873-07

**Authors:** Ch. Rauschenberg

**Affiliations:** Atlanta, Ga.


### ABSTRACTS FROM GERMAN JOURNALS.

BY CH. RAUSCHENBERG, M.D., ATLANTA, GA.

### *SUGGESTIONS IN RELATION TO THE TREATMENT OF CHOLERA.*

The distinguished Professor Botkin, of St. Petersburg (Russia), in an article contained in Nos. 33 and 34 of the *Berlin Klinical Weekly* (Zur Symptomatologie und Therapie der in Petersburg in Frühjahre, 1871, Beobachteten Cholera) publishes a series of observations made in his clinic on twenty-three cases of well developed cholera, all with a much weakened or entirely imperceptible pulse. He arrives at the conclusion that the intestinal affection characterizing the cholera process is not the consequence of the local effect of the cholera poison, but the expression of a general infection of the blood; that the cyanosis, dyspnœa, spasms, etc., are not the effect of an inspissation of the blood, but of the infection itself, which frequently causes death without producing diarrhœa. He concludes that a therapeutical action directed against the symptoms alone is inefficient, but that the general infection must be counteracted, and insists that quinine is the remedy which will most effectually resist the deleterious influences of the cholera poison on the human system. He administered this remedy in all the cases spoken of above, in five grain doses, two to four times daily. Where severe vomiting existed he gave it still oftener and generally hypodermically, and experienced only a mortality of 17.3 per cent. His favorite prescriptions are : ℞—Chinini muriat ʒ ss., acid muriat dil. gutt. xx; aquæ distil. ʒ v. M.—Sig. 15 drops, to be injected hypodermically several times daily, according to the severity of the case. Internally he uses at the same time: ℞ Tinct. chinini comp. spirit anodyne Hoffman ℥ ss., chinini mur. ʒ i., acid muriat dilut ʒ iss., olei menth. piper. gtt. x, sometimes with an addition of tinct. opii simpl. ʒ i, at 20 drops four to six times daily. In light cases he used the latter prescription alone.

Professor Botkin considers this treatment, according to his experience, quite worthy of consideration.

He also calls attention to the fact that in course of the winter preceding the cholera epidemic of which he is speaking, numerous cases of a morbid condition, with and without fever, were observed by him, and other physicians of that city, which, in their peculiar phenomena, showed a certain relationship to the cholera process, and appeared in epidemic diffusion. Diarrhœa, nausea, small pulse, profuse sweats, tenderness, swelling of the liver and spleen, scanty secretion of urine, sometimes with albuminuria, and in the convalescence herpes labialis, epistaxis, catarrhal affections of the nose, pharynx, and bronchia, and inclination to diarrhœa, were the prominent symptoms of these cases. They were successfully treated with quinine—five to six grains daily. Botkin looks upon this condition as an independent disease, but closely related to cholera, and probably caused by cholera poison modified, and changed by external circumstances—or, in other words, as a variety of cholera affection, an abortive form of the disease. He has also used in these cases—besides the quinine—with success: acid carbol cryst. gr. vi, chinini muriat ʒ i, extr. liquiri. q. s. fiat pilul No. 60. Sig.—Three pills twice daily.

His assistant, Dr. Popoff, injected substances vomited by cholera patients into the veins of dogs, and produced thereby *the clinical image of cholera, with the characteristic anatomical lesions of the intestinal canal.*

Lowndes (on the treatment of cholera, *Lancet*, September, 1871, page 319) recommends, from his long experience in India, in the stage of cholera diarrhœa, a mixture of from ten to fifteen drops of chloroform with the same quantity of tincture of opium, and three to four drachms of brandy in a glass of water, to be repeated if necessary. He insists upon the cautious use of opium in the algid stage; gives, internally, a few doses of calomel; applies counter-irritants externally, and gives freely of Liebig's beef tea, cold, as a beverage.

Schloeman (Die Behandlung der asiatischen Cholera durch das Schweflsaurc Chinin, *Berliner Klinische Wochenschrift*, Nos. 36 and 37, 1871,) has seen excellent effects from the quinine treatment in Antonio de Bejar, Western Texas, in all cases that had not already assumed a decided asphyxiated character. He administered in all cases of cholera-diarrhœa from seven to fifteen grains of sulph. quinine, at once, with about one grain of opium, and where vomiting existed, one hypodermic injection of three-tenths to one-half grain of morphia sulphas previously, and repeated the quinine, if light symptoms of quinine intoxication did not occur within a few hours. Where the diarrhœa did not cease soon, small doses of calomel were given every hour or every half hour.

The same favorable effects of the quinine treatment in cholera were observed by the late Dr. Stone, of New Orleans.

Dr. Von Kaczorowski, in charge of the City Hospital at Posen, in his very interesting report on the Cholera epidemic of 1866, (*Berliner Klinische Wochenschrift*, 1872, page 15, etc.,) of that city, expresses the opinion very confidently that the prognosis is decidedly more favorable in all cases where free vomiting and purging occurs. *He holds that the cholera poison is eliminated through the alimentary canal; that by its transfer into the blood, paralysis of the heart takes plaee, and that this transfer sets in more rapidly and fatally the sooner the alvine evacuations cease.* He noticed already during the cholera epidemics of 1852, '53, '55, and '56, in Poland, the favorable effect of evacuating remedies—for instance, of emetics of ipecacuanha and large doses of calomel, although many of the recovering patients had to endure a severe mercurial stomatitis. When castor oil was recommended from Berlin, in bloody cholera, he commenced the use of this mildest of all purgatives in the first desperate cases he met with, and the satisfactory results obtained by it encouraged him in its continued use. Castor oil and wine in repeated doses, with large doses of quinine occasionally during the stage of reaction, constituted from that time almost his entire treatment of cholera.

Every new patient, if in the early stages of cholera, was covered with a woolen blanket, his head and abdomen with cold cloths. Ice pills were administered freely, where a continued inclination to vomit prevailed, and one tablespoonful of castor oil, with tincture of camphor or oil of peppermint given immediately. When not retained, another dose was given at once, and the patient maintained in the horizontal position from fifteen to thirty minuutes, without being allowed to take any fluids during that time. In course of from one to two hours, generally, several discharges copiously mixed with flocculi (cast off intestinal epithelium) took place, and with them a perceptible amelioration of all symptoms. The restlessness and anxiety of the patient, the vomiting, rumbling and griping in the abdomen subsided, the cool extremities became warmer, and the small and frequent pulse slower and fuller.

Absolute abstinence from all ingesta, particularly food, during the first twelve hours; afterward one tablespoonsful or more of a strong astringent Hungarian wine, every hour or oftener, according to the weakness of the patient, during the next twelve hours, constituted the treatment, and was generally sufficient in the majority of erethic cases to abort the disease· Next day the patient received mucilaginous animal broths, and was generally discharged, well, on the third day.

If, as it frequently occurred, within a few hours after the first improvement, the abdominal troubles, and with them the restlessness and sinking of the pulse and temperature returned, another tablespoonful of castor oil was given, generally with the same effect as the first, and this course was continued until the development of gas within the abdomen ceased to take place.

Where, after these intestinal troubles, a peculiar oppression of the respiration, similar to that produced by coal oxyd gas poisoning (respiration about twenty per minute, inspiration superficial and short, expiration long and sighing, voice weak and hoarse, eyes bright, skin warm, covered with copious perspiration, face and extremities slightly livid, pulse perceptible to the last breath, temperature over 40° Cels, until a while after death) did not take place, the patient generally became convalescent at once, while, in cases already further advanced before treatment was commenced, the transition into the asphyctic stage, different from the first described condition, could often not be prevented. Where the patient already was in the algid stage when received, or got into it soon afterward, everything depended upon it whether he would survive the next few hours.

All external irritating applications or internal excitants prove useless for the purpose of restoring the marble-cold, livid extremities and surface of the body. Nothing is retained by the stomach, and resorption is so far reduced that even remedies hypodermically injected sometimes refuse to act. If this condition lasts any length of time, there is still a hope that the depressed action of the heart, only perceptible on the large vessels, may revive, and this hope increases if the checked secretions, particularly the alvine discharges, can be reëstablished. Therefore, even in this apparently hopeless collapse, the principal indication, Dr. Kaczorowski endeavored to fulfill is elimination of the poison, by the intestines, by giving again castor oil. The influence which this remedy has had in the restoration of his cases of that character he is not prepared to establish; but thinks it proper to call attention to the following facts in relation to it.

Castor oil was even in these cases the only remedy of which a part was retained, as the dejections, wherever they took place after giving it in such cases, contained numerous oil globules floating on their surface.

*Wherever flocculi and sometimes bile-like substances were discharged after giving the oil, a perceptible diminution of the restlessness of the patient and an improvement of skin and pulse took place.* Where the evacutions failed to appear soon, the paralyzed intestines were stimulated by an enema of spirits of turpentine with one-third of liquor ammoniæ. Wherever a slight improvement took place, frictions of the body with this mixture were made and strong wine was given every half hour, and this treatment continued until reaction was fully established. As often as this reaction vanished and algor and livor returned, the castor oil and the stimulating enema were repeated. If discharges of any size occurred, the flagging vitality would generally regain the ascendency, at least, for a time and thus this contest between life and death would often last three days. In these protracted asphyctic cases, bloody, violet-colored fluids, or rather fluids stained with livid blood, or gelatinous masses of mucus, or both, were discharged frequently, particularly the latter, with a remarkable decrease of the excessive tormina and tenesmus. Six patients of that character recovered under this treatment; many others entered the stage of reaction, but perished afterward with protracted dysenteric stools, under the symptoms of exhaustion.

As soon as the stage of reaction is fully established, frictions of the entire body, and castor oil as soon as the evacuations cease, are administered.

Dr. Von Kaczorowski does not look upon castor oil as a specific in this disease. He holds that the final result depends, principally, upon the degree of infection in each case, but that the proliferation and development of the cholera poison, which he thinks, under favorable circumstances, can and does take place within the human organism, and its entrance into the circulation from the macerated intestinal mucous membrane, can be interrupted and partly prevented by the expulsion of the poison from the alimentary canal.

This process of development occupies more or less time, according to his experience, as he has seen *no* case of cholera, where diarrhœa, vomiting, spasm, collapse, did appear altogether at once; but abnormal sensations in the abdomen, and diarrhœa, precede, in all cases, even if only a few hours, the real outbreak of the disease. Wherever, during a cholera epidemic, these premonitory symptoms occur, he deems it urgently necessary to empty the bowels. Since he has observed this rule he has not seen a single case in which these premonitory symptoms have developed themselves into a severe, much less a fatal condition.

Thus he looks upon *castor oil as the most reliable remedy for the rapid removal of the prodroma of cholera, and a most valuable preventive of the severe forms of the disease.*

Dr. John Murray, Inspector-General of Hospitals, in the Medical Department of India, received from three hundred and fifty physicians, who used castor oil in the commencement of cholera in that country, favorable reports, while five hundred and sixty-five condemned the use of opium in the algid stage.

It is the firm conviction of the writer of this article that all specific contagious, infectious, and miasmatic diseases—such as variola, scarlatina, measles, typhus, cholera, pyæmia, puerperal fever, yellow fever, malarial fevers, etc.—are *the distinct pathological effect of just as specific substances in nature as these diseases themselves are specific*, and that the continued search after them in the juices and excretions of the diseased human system, with the microscope, will gradually remove the mystic darkness still prevailing, and bring their nature to light, as it has already done, to a considerable extent, in relation to the instigators of seveial specific diseases of the skin, of pyæmia, of pustula maligna, of diphtheria, and of variola.*

There can be no doubt that many of these invisible instigators of specific diseases, as the microscope has correspondingly revealed, *must be protoplasma—living substance, living beings—because such only can possess the power of germination, growth, reproduction and multiplication, which they so plainly exhibit.* Those of them that develop themselves, and exercise this power of reproduction alone within the human system, seem to possess it in the highest degree,—contagion of variola, scarlatina, measles, etc.; others, while they perhaps regenerate themselves in a very different manner within the human system, pass through it without losing their specific vitality, and power of germination and reproduction, but continue to live, grow, and multiply under favorable external conditions, (cholera poison, typhus poison, etc.); still others do not seem to possess the power of germination, reproduction, and transfer from individual to individual—for instance, malaria—and they must either represent unorganized chemical compounds, or at least organisms which lose their vitality within the human system.†

There can be but little doubt that the cholera poison must have a life of its own; that it must be organized substance of a vegetable or animal nature, and that in all probability it is closely related to the many known microscopic organisms

* Microscopic Organisms as the Instigators of Disease. *Atlanta Medical and Surgical Journal*, Sept. 1872.

† Uber Enstehung u Verbreitnng des Abdominal Typhus. Volkmanns Samlung Klin. Vortrage, No. 53, February 1873.

which, principally as instigators of fermentation, decomposition, and sepsis, (pencillium, vibrio, bacterium, etc.,) play an important part in the production of specific diseases. If this at least very reasonable hypothesis is correct, the usefulness of quinine in the treatment of cholera, becomes all at once very evident, as Binz, in 1868, furnished direct experimental proof, that quinine acts specifically poisonous on a large majority of low vegetable and animal organisms and their germination, nutrition and reproduction, even when used in very minute portions. Thus quinine becomes an internal disinfectant and antiseptic par excellence, and its use in cholera, for the purpose of checking the development of the cholera germ within the human system, is in perfect accordance with the latest views on its therapeutical powers.

Since Hallier's and Klob's investigations have demonstrated the presence of innumerable spores of microscopic fungi in the dejections of cholera patients, since Thiersche's and Popoff's experiments on animals have proven that these substances, when introduced into the stomach, or the circulation, produce the disease in those animals; and since it has become an acknowledged fact that the dejections of cholera patients contained in soiled articles of clothing, in the chamber vessels and privies are the only bearers of the cholera poison, and the centers of infection from which cholera epidemics originate, the opinion that the stomach and intestinal canal are the localities of the human system where the most favorable conditions for the reception and more or less rapid development of the cholera germ exist, and where, therefore, the poison makes its first and its most intense morbid impression, and from where it continues to effect the entire system, or to enter the circulation during the whole course of the disease has been almost generally accepted.

Thus the opinion of Dr. Kaczorowski that frequent vomiting, and particularly purging, relieves the patient by diminishing the quantity of the poison engaged in a process of rapid development and multiplication, and thus lessening the degree and duration of the general infection finds theoretical support. If, however, as Niemeyer and others hold, the highest grades of the cholera process, the depression of the action of the sympathetic, the paresis of the heart, the asphyctic condition with its terrible collapse are the direct consequences of the extensive epithelial abrasion of the small intestines, of the subsequent enormous transudation of blood serum, and inspissation of the blood, the idea that a continuation of this abundant discharge should be attended with favorable consequences loses, apparently, its force, and the evacuating treatment of Dr. Kaczorowski appears at first sight of very doubtful propriety. If it is correct, however, that the severe and extensive local lesion of the intestinal mucous surface, with its subsequent depression on the sympathetic, its excessive transudation of blood serum, imperfect circulation in the capillaries generally and in those of the heart in particular, and the paresis of the heart, thus initiated, is primarily caused *by the presence of the cholera germ in the intestinal canal*, the continued removal of that poison, in Dr. Kaczorowski's sense, must necessarily be an important if not the principal indication during the entire duration of the disease, particularly if the opinion that the cholera germs there are engaged in a process of rapid development and multiplication (fermentation) be correct.

Dr. C. F. Kunze, of Halle, remarks in his late work on Practical Medicine, in correspondence with Dr. Kaczorowski's observations that the copious alvine discharges generally give relief to the patient; and Niemeyer, a strong advocate of the opinion that the excessive transudation from the intestinal mucous surface is the cause of the great danger and fatality of the disease, admits that the cessation of the watery discharges is an unfavorable prognostic sign, but, according to his views, not because it is an evidence of checked transudation, but of paralysis of the muscular coat of the intestines.

---

At a meeting of the Medical Society of Victoria, held in June, 1872, the following collection, taken from the stomach of a lunatic upon whom a post mortem examination had been made, was exhibited: Twenty-five pebbles, sixteen pieces of glass, twelve nails, one screw, two bones, a button, five bits of tin, a peach stone, six fragments of a pipe stem, and a whole cutty pipe.

